# Cutoff Values of Serum IgG4 and Histopathological IgG4+ Plasma Cells for Diagnosis of Patients with IgG4-Related Disease

**DOI:** 10.1155/2012/580814

**Published:** 2012-05-10

**Authors:** Yasufumi Masaki, Nozomu Kurose, Motohisa Yamamoto, Hiroki Takahashi, Takako Saeki, Atsushi Azumi, Shinji Nakada, Shoko Matsui, Tomoki Origuchi, Susumu Nishiyama, Kazunori Yamada, Mitsuhiro Kawano, Akira Hirabayashi, Keita Fujikawa, Tomoko Sugiura, Masanobu Horikoshi, Naoto Umeda, Hiroshi Minato, Takuji Nakamura, Haruka Iwao, Akio Nakajima, Miyuki Miki, Tomoyuki Sakai, Toshioki Sawaki, Takafumi Kawanami, Yoshimasa Fujita, Masao Tanaka, Toshihiro Fukushima, Katumi Eguchi, Susumu Sugai, Hisanori Umehara

**Affiliations:** ^1^Hematology and Immunology, Kanazawa Medical University, 1-1 Daigaku, Uchinada, Kahoku-gun, Ishikawa 920-0293, Japan; ^2^Department of Pathology and Laboratory Medicine, Kanazawa Medical University, 1-1 Daigaku, Uchinada, Kahoku-gun, Ishikawa 920-0293, Japan; ^3^First Department of Internal Medicine, School of Medicine, Sapporo Medical University, S1 W17 Chuo-ku, Sapporo, Hokkaido 060-8556, Japan; ^4^Department of Internal Medicine, Nagaoka Red Cross Hospital, Sensyu-2 297-1, Nagaoka-shi, Niigata 940-2085, Japan; ^5^Ophthalmology Department, Kobe Kaisei Hospital, Shinohara Kitamachi 3-11-15, Nada Ku, Kobe City, Hyogo 657-0068, Japan; ^6^Department of Japanese Oriental Medicine, University of Toyama, 2630 Sugitani, Toyama city, Toyama 930-0194, Japan; ^7^First Department of Internal Medicine, University of Toyama, 2630 Sugitani, Toyama city, Toyama 930-0194, Japan; ^8^Nagasaki Graduate School of Health Sciences, 1-7-1 Sakamoto, Nagasaki city, Nagasaki 852-8501, Japan; ^9^Rheumatic Disease Center, Kurashiki Medical Center, 250 Bakuro-chou, Kurashiki, Okayama 710-8522, Japan; ^10^Division of Rheumatology, Department of Internal Medicine, Kanazawa University, 13-1 Takara-machi, Kanazawa, Ishikawa 920-8641, Japan; ^11^Hiroshima Bay Clinic, 2-2-19 Yanoshin-machi, Aki-ku, Hiroshima city, Hiroshima 736-0084, Japan; ^12^Department of Internal Medicine, Isahaya General Hospital, 24-1 Eishohigasi-macki, Isahaya, Nagasaki 854-8501, Japan; ^13^Sugiura Clinic, 2-8-3 Imaichi-chou, Kitashin-machi, Izumo, Shimane 693-0002, Japan; ^14^Department of Rheumatology, University of Tsukuba, 2-1-1 Amakubo, Tsukuba, Ibaraki 305-8576, Japan

## Abstract

IgG4-related disease is a new disease classification established in Japan in the 21st century. Patients with IgG4-related disease display hyper-IgG4-gammaglobulinemia, massive infiltration of IgG4+ plasma cells into tissue, and good response to glucocorticoids. Since IgG4 overexpression is also observed in other disorders, it is necessary to diagnose IgG4-related disease carefully and correctly. We therefore sought to determine cutoff values for serum IgG4 and IgG4/IgG and for IgG4+/IgG+ plasma cells in tissue diagnostic of IgG4-related disease. *Patients and Methods*. We retrospectively analyzed serum IgG4 concentrations and IgG4/IgG ratio and IgG4+/IgG+ plasma cell ratio in tissues of 132 patients with IgG4-related disease and 48 patients with other disorders. *Result*. Serum IgG4 >135  mg/dl demonstrated a sensitivity of 97.0% and a specificity of 79.6% in diagnosing IgG4-related disease, and serum IgG4/IgG ratios >8% had a sensitivity and specificity of 95.5% and 87.5%, respectively. IgG4+cell/IgG+ cell ratio in tissues >40% had a sensitivity and specificity of 94.4% and 85.7%, respectively. However, the number of IgG4+ cells was reduced in severely fibrotic parts of tissues. *Conclusion*. Although a recent unanimous consensus of all relevant researchers in Japan recently established the diagnostic criteria for IgG4-related disease, findings such as ours indicate that further discussion is needed.

## 1. Introduction

IgG4-related disease (IgG4-RD), a new disease classification first established in Japan in the 21st century, is characterized by hyper-IgG4-gammaglobulinemia and massive infiltration of IgG4-positive plasma cells into various swollen organs [1–10]. In general, a serum IgG4 concentration >135 mg/dL has been established as the cutoff value for the diagnosis of patients with IgG4-RD and is used in the joint consensus criteria of the Okazaki and Umehara groups investigating IgG4-RD for the Ministry of Health, Labor, and Welfare of Japan [11].

Some patients with early or limited stage IgG4-RD, however, may show the sufficient pathological characteristics and clinical features of this disease, such as good response to glucocorticoids, despite having serum IgG4 concentrations <135 mg/dL. In addition, an IgG4+/IgG+ ratio >40% in tissue and >10 cells/high-power field (HPF) have been used in the histopathologic diagnosis of IgG4-RD in Japan. Thus, a proper diagnosis of these patients may require the use of other criteria, including IgG4+/IgG plasma cell ratio. Since lower cutoff values may increase sensitivity while decreasing specificity, it is necessary to establish accurate cut off values for this ratio.

To better establish the diagnostic criteria for IgG4-RD, we, the members of the IgG4+MOLPS/Mikulicz's disease research group in Japan, sought to determine the cutoff values for serum IgG4 and IgG4/IgG and for IgG4+/IgG+ plasma cells in tissue diagnostic of IgG4-RD using retrospectively collected data.

## 2. Materials and Methods

### 2.1. Measurement of Serum IgG4 Concentration

Serum IgG4 concentrations and IgG4/IgG ratio and the ratio of IgG4+/IgG+ plasma cells in tissue were determined in 132 patients with IgG4-RD and 48 patients with other disorders registered retrospectively in the IgG4+MOLPS/Mikulicz's disease research group (Table 1). The 48 patients with other disorders included 33 with Sjögren's syndrome, 3 with multicentric Castleman's disease (MCD), 3 with B-cell lymphoma, 2 with sarcoidosis, and 1 each with Kimura's disease, ulcerative colitis, autoimmune hepatitis, IgG-type monoclonal gammopathy of undetermined significance, progressive transformation of the germinal center, scleritis, and severe keratoconjunctivitis sicca. The study was approved by the review boards of Kanazawa Medical University and all other collaborating institutions, and all patients provided written informed consent for the use of their data and samples. The sensitivities, specificities, and ROC curves of serum IgG4 >135 mg/dL and various serum IgG4/IgG ratios were statistically analyzed using SPSS v.11 (SPSS Inc., Chicago, IL, USA).

Patients with borderline IgG4-RD were defined carefully as those with (1) >40% IgG4+/IgG+ plasma cells in tissue, (2) strict pathological differential, and (3) a typical clinical course (spontaneous regression or no change without treatment, or good response to an initial daily dose of <0.6 mg/kg prednisolone).

### 2.2. Analysis of IgG4+ Cells in Tissue

The numbers of IgG4+ and IgG+ cells in tissue samples from 36 patients with IgG4-RD and from 21 with other disorders were determined by counting cells counts in 5 high-power fields (HPF) under light microscopy. We also recounted cell areas of 17 samples from patients with IgG4-RD that contained both fibrotic and nonfibrotic parts.

We also assessed the sensitivity and specificity of IgG4+/IgG+ cell ratios >10%, >20%, >30%, >40%, and >50%, and of >10, >20, >30, >40 and >50 IgG4+cells/HPF, as well as the presence of obliterative phlebitis, storiform fibrosis, eosinophilia, fibrosis, and lymphocyte infiltration as determined by hematoxylin and eosin staining in the diagnosis of IgG4-RD.

Tissue samples with borderline IgG4-RD were defined as those with (1) serum IgG4 >135 mg/dL, (2) strict pathological differential, and (3) typical clinical course. Clinical course including response to steroid is not included in the comprehensive diagnostic criteria for IgG4-RD, to avoid the needless treatment with steroid of patients suspected of having IgG4-RD. As this was a retrospective analysis, however, we analyzed the clinical course of these borderline patients, including their response to steroid treatment.

## 3. Results

### 3.1. Serum IgG4 Concentration

A serum IgG4 cutoff value >135 mg/dL had a sensitivity of 97.0% and a specificity of 79.6% for the diagnosis of IgG4-RD. In 4 patients with relatively small and restricted lesions, however, this criterion was not adequate to diagnose IgG4-RD, although all had a histopathology and clinical course compatible with IgG4-RD (Table 2). All 4 patients were diagnosed with IgG4-RD based on a serum IgG4/IgG ratio >8%.

**Figure 1:**
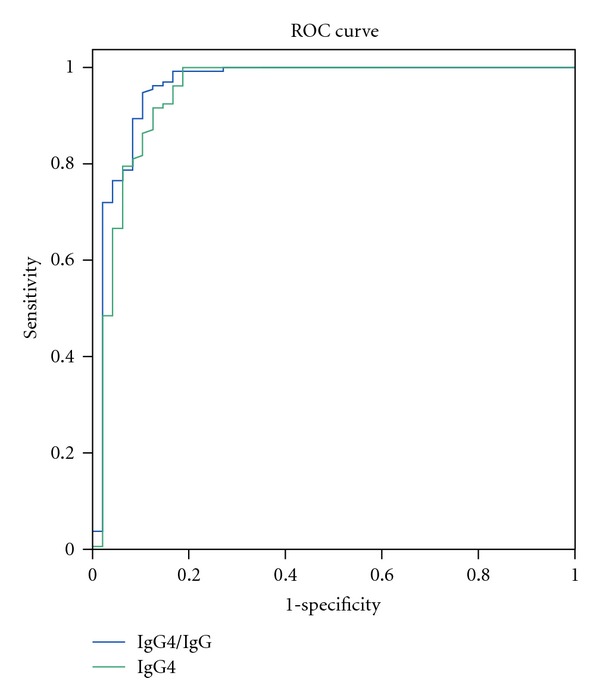
ROC curves of absolute serum IgG4 concentration (green) and serum IgG4/IgG ratio (blue). The two curves were almost identical.

In contrast, neither a serum IgG4 cutoff of >135 mg/dL nor a serum IgG4/IgG ratio >8% was adequate for the diagnosis of four patients with MCD and one each with B-cell lymphoma, scleritis, and Sjögren's syndrome, because all of these patients had hyper-IgG4-globulinemia associated with polyclonal gammopathy. We therefore estimated the sensitivity and specificity of various IgG4/IgG ratios (Table 3). We found that the ROC curves for absolute serum IgG4 concentration and serum IgG4/IgG ratio were almost identical (Figure 1).

### 3.2. Analysis of IgG4+ Cells in Tissue Samples

We also assessed the ability of the ratio of IgG4+/IgG+ plasma cell ratios in 5 HPFs of tissue samples to diagnose IgG4-RD. We found that a ratio >40% had a sensitivity of 94.4% and a specificity of 85.7% (Table 4). Although >10 IgG4+ cells per HPF had a sensitivity of 100%, They had specificities of only 38.1%.

In tissues containing both fibrotic and nonfibrotic areas, we counting the number cells in each part showed that fibrotic areas contained fewer IgG4+ cells (Table 5).

We also assessed the ability of obliterative phlebitis and storiform fibrosis to diagnose IgG4-RD. Although both had specificities of 100%, their sensitivities were not very high (Table 4).

## 4. Discussion

**Figure 2:**
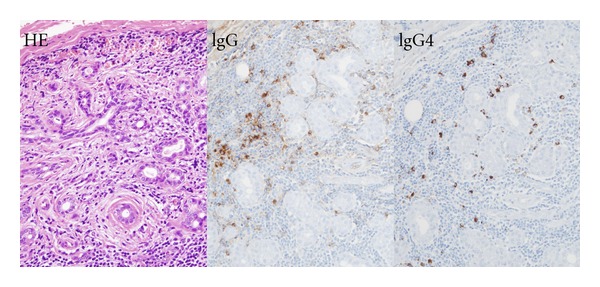
Histopathology of a patient with limited stage IgG4-related dacryoadenitis. A 35-year-old woman presented with swelling of her bilateral lacrimal glands. Her serum IgG and IgG4 concentrations were 1,149 mg/dL and 125 mg/dL, respectively, and her serum IgG4/IgG ratio was 10.88%. Histopathological examination of a lacrimal gland biopsy revealed IgG4+ plasma cell infiltration, with an IgG4+/IgG+ cell ratio of 74.7%, and 53 IgG4+ cells per HPF, although sclerotic changes were not severe. Treatment with prednisolone 20 mg/day resulted in a rapid and dramatic improvement in symptoms. Although using an absolute serum IgG4 cutoff concentration of >135 mg did not result in diagnosis of IgG4-RD, her clinical course and histopathology were typical of IgG4-RD. However, using a serum IgG4/IgG ratio >8% as a cutoff value resulted in a diagnosis of IgG4-RD.

Serum IgG4 >135 mg/dL has been widely accepted as a cutoff value for diagnosis of IgG4-RD. Although this concentration was determined by comparing patients with IgG4-related sclerosing pancreatitis and those with pancreatic cancer [1], it has also been used to diagnose IgG4-RD involving other organs. For example, we have utilized this cutoff value as a diagnostic criterion for IgG4-related Mikulicz's disease [5] and IgG4-related kidney disease [12], and, in 2011, it was adopted in the comprehensive clinical diagnostic criteria of IgG4-related diseases [11]. Most patients with IgG4-RD show multiple organ involvement at diagnosis, with both high absolute serum IgG4 concentrations and serum IgG4/IgG ratios. However, some patients with early and/or limited stage IgG4-RD do not present with high IgG4-globulinemia (Figure 2), with some not having IgG4 concentrations >135 mg/dL. We have therefore tested the ability of alternative criteria to diagnose for IgG4-RD. Although we found that a serum IgG4/IgG ratio >5% had the highest sensitivity, the normal ratio is about 5-6%, making this cut off value misleading. An IgG4/IgG ratio >8% had a sensitivity similar to that of absolute IgG4 >135 mg/dL, but a greater specificity, enabling us to diagnose 4 patients with lower absolute IgG4 concentrations as having IgG4-RD (Table 2). Since the standard cut off of absolute IgG4 >135 mg/dL demonstrated excellent sensitivity and specificity, it should be utilized, except for patients with early and/or limited IgG4-RD, for whom we propose using an IgG4/IgG ratio >8%.

Careful diagnosis is required in patients with lower IgG4 concentrations, since those patients may have other distinct disorders with different clinical features than IgG4-RD. Patients with untypical clinical courses, including glucocorticoid refractoriness, should be reassessed.

IgG4+/IgG+ plasma cell ratios in tissue >40% and >50%, and >10 IgG4+ cells per HPF have been used for the diagnosis of IgG4-RD. We found that an IgG4+/IgG+ cell ratio >40% in tissue had a sensitivity of 94.4% and a specificity of 85.7% in the diagnosis of IgG4-RD. We also found that IgG4+ plasma cell concentrations in tissue were diminished in fibrotic tissue areas, suggesting that a ratio >40% is a better histopathologic cutoff value. The presence of obliterative phlebitis and storiform fibrosis demonstrated specificities of 100%, but their sensitivities were much lower, indicating that these findings would be useful when added to, but not in place of, other results.

Since patients with disorders such as MCD and lymphoma may demonstrate hyper-IgG4-gammaglobulinemia and massive IgG4+ plasma cell infiltration in tissue, serum IgG4 concentration and IgG4+ cells in tissue are not specific indicators of IgG4-RD. Rather, a diagnosis of IgG4-RD should be based on the overall balance of clinical features, such as disease distribution throughout the body, clinical course, serum concentrations, and histopathology.

The pathologic consensus statement of the first international Symposium on IgG4-RD in Boston did not adopt IgG4+/IgG+ cell ratio in tissue as diagnostic, although it did suggest cutoffs for numbers of IgG4+ cells in HPFs of various organs. This, however, may be confusing for many pathologists and physicians. Although pathologic findings are very important in the diagnosis for IgG4-RD, clinical features and serological findings should be included.

Recently, some patients with IgG4-RD were found to have lymphoma [13, 14] and other types of cancer [15, 16]. Thus IgG4-RD may not always be a benign disease with good prognosis. Many patients referred to our centers with glucocorticoid refractory IgG4-RD were diagnosed incorrectly, suggesting the need for more accurate diagnostic criteria for these diseases.

## Figures and Tables

**Table 1:** Involved organs in 132 patients with IgG4-RD. More than half (64 cases) the patients in our series had so-called Mikulicz's disease, with symmetrical swelling of at least two sets of lacrimal, parotid, or submandibular glands.

Involved organ	Number of patients
Total	132

Lacrimal gland	75
Parotid gland	32
Submandibular gland	74
Pancreas	20
Kidney	21
Lung	6
Lymph node	4
Thyroid	4
Liver	3
Retroperitoneum	3
Dura	1
Hypophysis	1
Bile duct	1

**Table 2:** Serum IgG and IgG4 concentrations and IgG4/IgG4 ratio of patients with false-positive and false-negative diagnoses of IgG4-RD.

False positives			

Diagnosis	IgG (mg/dL)	IgG4 (mg/dL)	IgG4/IgG

(1) MCD	7,080	4,560	64.4%
(2) MCD	4,420	690	15.6%
(3) B-cell lymphoma	2,510	456	18.2%
(4) MCD	2,960	295	10.0%
(5) MCD	2,476	295	11.9%
(6) B-cell lymphoma	4,010	271	6.8%
(7) Scleritis	2,900	232	8.0%
(8) Sarcoidosis	1,380	189	13.7%
(9) Sjögren's syndrome	3,920	171	4.4%
(10) Malignant lymphoma	1,930	141	7.3%

False negatives			

Diagnosis	IgG(mg/dL)	IgG4(mg/dL)	IgG4/IgG

(1) IgG4-RD	1,149	125	10.9%
(2) IgG4-RD	1,210	123	10.2%
(3) IgG4-RD	1,228	111	9.0%
(4) IgG4-RD	1,260	106	8.4%

IgG4-RD: IgG4-related disease; MCD: multicentric Castleman's disease.

**Table 3:** Sensitivity and specificity of serum cutoff values in the diagnosis of IgG4-RD.

	Sensitivity	Specificity
IgG4 > 135 mg/dL	97.0%	79.6%
IgG4/IgG > 5%	99.2%	83.3%
IgG4/IgG > 6%	97.0%	83.3%
IgG4/IgG > 7%	97.0%	85.4%
IgG4/IgG > 8%	95.5%	87.5%
IgG4/IgG > 9%	92.4%	89.6%
IgG4/IgG > 10%	89.4%	91.7%

**Table 4:** Sensitivity and specificity of pathological findings for the diagnosis of IgG4-RD.

	Sensitivity	Specificity
IgG4+/IgG+ > 10%	100.0%	33.3%
IgG4+/IgG+ > 20%	100.0%	47.6%
IgG4+/IgG+ > 30%	100.0%	71.4%
IgG4+/IgG+ > 40%	94.4%	85.7%
IgG4+/IgG+ > 50%	94.4%	95.2%
IgG4+cells/HPF > 10	100.0%	38.1%
IgG4+cells/HPF > 20	97.2%	42.9%
IgG4+cells/HPF > 30	97.2%	61.9%
IgG4+cells/HPF > 40	91.7%	66.7%
IgG4+cells/HPF > 50	86.1%	71.4%
Obliterative phlebitis	54.5%	100.0%
Storiform fibrosis	31.4%	100.0%
Eosinophilia	42.9%	100.0%
Fibrosis	91.4%	82.4%
Lymphocytic infiltration	100.0%	16.7%

**Table 5:** Recounting in each areas of 17 samples containing both fibrotic and nonfibrotic parts. All patients were diagnosed with IgG4-RD but had biopsy specimens that were too small (samples 1–14) or with relatively large fibrotic areas inadequate to diagnose IgG4-RD (samples 15–17). All samples had >10 IgG4+ cells per HPF.

Tissue	Fibrosis+	Fibrosis−
(1) Pancreas	76.6%	94.0%
(2) Submandibular gland	75.4%	89.2%
(3) Submandibular gland	72.1%	73.0%
(4) Submandibular gland	72.1%	97.3%
(5) Submandibular gland	71.8%	99.1%
(6) Pancreas	67.7%	95.0%
(7) Labial salivary glands	65.0%	70.8%
(8) Lung	58.9%	94.4%
(9) Submandibular gland	49.2%	68.9%
(10) Gall bladder	48.6%	94.0%
(11) Bile duct	46.8%	95.0%
(12) Submandibular gland	46.2%	74.1%
(13) Orbit	44.2%	94.4%
(14) Submandibular gland	43.6%	95.0%
(15) Submandibular gland	33.3%	95.0%
(16) Submandibular gland	25.9%	51.5%
(17) Labial salivary glands	8.0%	76.2%
